# Enabling Continuous Quality Improvement in Practice: The Role and Contribution of Facilitation

**DOI:** 10.3389/fpubh.2017.00027

**Published:** 2017-02-22

**Authors:** Gillian Harvey, Elizabeth Lynch

**Affiliations:** ^1^Adelaide Nursing School, University of Adelaide, Adelaide, SA, Australia; ^2^Alliance Manchester Business School, University of Manchester, Manchester, UK; ^3^Florey Institute of Neuroscience and Mental Health, Parkville, VIC, Australia

**Keywords:** facilitation, facilitators, continuous quality improvement, implementation, context

## Abstract

Facilitating the implementation of continuous quality improvement (CQI) is a complex undertaking. Numerous contextual factors at a local, organizational, and health system level can influence the trajectory and ultimate success of an improvement program. Some of these contextual factors are amenable to modification, others less so. As part of planning and implementing healthcare improvement, it is important to assess and build an understanding of contextual factors that might present barriers to or enablers of implementation. On the basis of this initial diagnosis, it should then be possible to design and implement the improvement intervention in a way that is responsive to contextual barriers and enablers, often described as “tailoring” the implementation approach. Having individuals in the active role of facilitators is proposed as an effective way of delivering a context-sensitive, tailored approach to implementing CQI. This paper presents an overview of the facilitator role in implementing CQI. Drawing on empirical evidence from the use of facilitator roles in healthcare, the type of skills and knowledge required will be considered, along with the type of facilitation strategies that can be employed in the implementation process. Evidence from both case studies and systematic reviews of facilitation will be reviewed and key lessons for developing and studying the role in the future identified.

## Introduction

Quality improvement interventions are typically complex and multifaceted, encompassing both technical and social components (1). As such, whether and how well they work depends on the dynamic interplay between the components of the improvement interventions, the people involved, and the organizational and health system context in which they work. Understanding and managing these complex interactions presents a challenge to those charged with the responsibility for designing, planning, implementing, and evaluating quality improvement initiatives in the real world (2).

With the growing science of healthcare improvement, knowledge has advanced in a number of key areas. First, there is a greater understanding of the components to combine together within an improvement intervention. For example, activities centered around audit and feedback, goal setting, and the use of Plan-Do-Study-Act (PDSA) cycles can help to establish a clear need for improvement, guide the direction of travel, and enable progress toward agreed aims to be monitored (1, 3). Second, our understanding of the influence of contextual factors on the way that improvement programs play out in practice has increased substantially (4). Various frameworks have been developed and applied to help assess and make sense of these contextual factors at a program, organizational, and wider system level (5, 6). Third, acknowledging the complexity of implementation and the fact that “context matters” (7), the need to tailor improvement interventions accordingly is recognized (8). This raises an important question—and one that we address in this paper—namely, how best to achieve effective facilitation of improvement interventions that are tailored to take account of the specifics of the improvement project, the contextual setting, and the individuals and teams involved.

## Tailoring to Context—Who and How?

Some contextual factors are amenable to modification, whereas other factors must be identified and “worked around.” Typically, factors within the inner context, such as the extent of local leadership support or employee motivation to engage in improvement, can be addressed with specific strategies. However, wider contextual factors at the health system or policy level need to be acknowledged as influential, but are unlikely to be amenable to change. Therefore, as part of planning and implementing an improvement intervention, it is important to assess and build an understanding of the contextual factors that are likely to influence implementation, both positively and negatively. This includes looking at factors such as the organizational culture and receptiveness to new ideas and change; what the past experience of improvement has been; whether leaders (clinical and executive) are supportive; how much authority people have to make decisions and introduce new ideas; and whether resources will be available to support the introduction of the proposed improvements. On the basis of this initial assessment, it should then be possible to design and implement the intervention in a way that is responsive to contextual barriers and enablers—or, in other words, to “tailor” the improvement program. However, even with prior knowledge and understanding, achieving effective tailoring in practice can be challenging (2). Having some form of human component within the process is seen as beneficial to actively manage the barriers and enablers that are encountered (9). Various roles are described in the implementation and improvement literature, for example, knowledge brokers and boundary spanners (10), opinion leaders (11), academic detailers (12), improvement coaches (13, 14), and educational outreach visitors (15), to name just a few. While terminology varies and often overlaps, there are some distinct differences in terms of how specific roles function (16). For example, opinion leaders typically operate through peer influence and being accepted as a credible expert; academic detailers employ social marketing techniques; improvement coaches emphasize goal setting and self-reflection; and educational outreach encompasses learning processes.

Given the complexities associated with quality improvement and the challenge of tailoring improvement interventions appropriately to context, the question could be which of the roles described above are the best ones to employ in which particular setting or situation? Or is it more a case of knowing which combination of roles to bring together and how? In attempting to address these questions, we draw on our own experiences of applying facilitation roles and processes in quality improvement in healthcare, supported by empirical evidence from the literature. We begin by defining what we mean by facilitation and how it relates to other roles that support quality improvement. We then consider how facilitator roles can be employed in quality improvement initiatives and what existing research can tell us about the effectiveness of the role and key issues to consider. We conclude by outlining what we see as fruitful questions for further study.

## Defining Facilitation and the Facilitator Role

Taken literally, the word facilitation means to “make easier.” As a concept, facilitation began to emerge in healthcare in the latter half of the twentieth century, influenced by humanistic principles of participation, engagement, and shared decision-making and enabling others (17). This philosophy aligns well with the principles underpinning continuous quality improvement (CQI), influenced by the early work of theorists such as Deming and Juran, who emphasized the importance of employees taking responsibility for quality, rather than being subject to performance assessment and inspection by managers (18).

Facilitator roles in primary healthcare improvement are evident from the 1980s onward, supporting a range of initiatives in screening and prevention [see, for example, Ref. (19–21)]. Our own experience of facilitation and facilitator roles similarly has its roots in quality improvement (22), and more recently, in the related field of knowledge translation and implementing evidence-based healthcare (23, 24). In the widely used Promoting Action on Research Implementation in Health Services framework, facilitation has been defined as the active ingredient that aligns the proposed innovation or improvement to the individuals and teams involved and the context in which they work, thereby enabling successful implementation (25). In order to operationalize facilitation, it is important to develop and support individuals in facilitator roles to act as the human agent in implementation, whether they are facilitating a new innovation in practice, the implementation of evidence-based clinical guidelines or agreed goals for practice improvement (25).

A question frequently posed is “who can take on the facilitator role”? The quick answer is that there is no single job specification. Facilitators can be internal or external to the organization, from a clinical or non-clinical background, and be operating at different organizational levels from a clinical team through to the wider health system level. The key is that they meet the requirements of the role, in terms of their personal attributes, knowledge, and skills. Commonly described personal characteristics of facilitators include being empathetic, sensitive, flexible, pragmatic, authentic, credible, resilient, and passionate (26, 27). Alongside these individual attributes, typical skills required include a mix of technical and process skills as facilitators characteristically occupy a hybrid role, balancing the achievement of improvement goals with the development of effective teamwork processes and building improvement capacity in individuals. This requires skills in project management and improvement methods, combined with skills in interpersonal communication, group processes, negotiation, and empowering others (28). It is increasingly clear that the role—and the range of skills and knowledge required—is typically too complex for lone individuals to take on and that in order to manage large improvement programs, a network of facilitators with varying levels of expertise and responsibility is required (16).

## How is Facilitation Different to Other Roles?

The literature illustrates many examples of facilitators working in different settings on a wide range of different improvement initiatives. These include improving the management of nutrition in hospital (29), reducing neonatal mortality in a rural community (30) and improving chronic disease management in primary care (31), to name just a few examples. The main distinguishing feature of the role is the focus on enabling, as opposed to persuading, influencing, directing, or coercing. The facilitator does not control or mandate what needs to be done, but rather helps the individuals in an improvement team to work collaboratively to agree areas for improvement and create and sustain change in healthcare provision. Clearly there may be overlap with other change agent roles and a close working relationship with formal and informal leaders is essential (32); however, the facilitator typically takes on a more generic, coordinating role that involves working with and through others to achieve improvement. In turn, this helps to embed capacity for change within teams and organizations by influencing workplace culture, and empowering and upskilling team members to facilitate change (33, 34).

## How Do Facilitators Enact the Role?

In fulfilling a role that involves enabling teams to achieve their improvement goals, the facilitator employs a range of facilitation methods and processes. Some of these are more directly concerned with the improvement task (for example, setting goals and agreeing audit measures); others focus on managing the process (for example, establishing ground rules for the improvement team, responding to contextual barriers, and managing conflict). Facilitation is an iterative process, but comprises a number of core elements. A typical facilitator’s “toolkit” includes attention to issues such as clarifying and engaging stakeholders; undertaking a baseline assessment; planning and implementing; and reviewing, sharing, and recognizing success (35). Table 1 summarizes the type of activities that the facilitator is likely to be engaged in to address these issues.

**Table 1:** **Example facilitation strategies [adapted from Ref. (35)]**.

Area of focus	Key activities
Clarifying and engaging	Identifying and clarifying the improvement issue to be addressed Establishing the level of interest and commitment to the improvement topic Identifying local champions and wider stakeholders Getting the right people together to form an improvement team Developing a preliminary project plan Securing stakeholder support
Assessing and measuring	Developing an understanding of the state of “readiness”—motivation and capability to be involved in the proposed improvement Baseline assessment of individuals, teams, and contextual barriers and enablers Undertaking baseline audit
Action and implementation	Review and interpretation of baseline data Developing an agreed implementation and action plan Running small tests of change (Plan-Do-Study-Act cycles) Tracking progress over time and adapting as required
Reviewing and sharing	Undertaking repeat audit Reflecting on the process: what worked well and less well Feeding back to wider stakeholder group Organizing a “celebratory event” Planning for sustainability and spread

## What Evidence is There for Facilitation?

Baskerville and colleagues undertook a systematic review of practice facilitation in primary care. From analysis of the 23 included studies, the review concluded that practices supported by facilitators were 2.76 times more likely to adopt evidence-based clinical guidelines (36). In all 23 studies, facilitators used audit and feedback as part of their implementation strategy. Other facilitation strategies included interactive consensus building and goal setting, reminder systems, tailoring to context, and the use of improvement tools such as PDSA. Analysis also indicated that tailoring the interventions to the context and needs of the practice led to significantly more positive effects when compared to studies that did not tailor the interventions. When facilitation interventions were provided at higher intensities (more frequently and/or for longer periods of time), the effectiveness was significantly greater.

More recent evidence supporting the impact of facilitation comes from a large cluster-randomized trial in Vietnam. Intervention groups received support from trained lay workers as facilitators of quality improvement in community groups and demonstrated a significant reduction in neonatal mortality after 3 years (37). This study also highlighted the importance of the skills of the facilitator, demonstrating that those teams that received the highest level of facilitation (as judged by a rating of facilitator skills and effectiveness) achieved greater improvements in neonatal mortality (30).

As facilitation is becoming more commonly applied and evaluated in healthcare quality improvement and implementation, then the knowledge base will continue to grow. For example, studies are reported in the literature comparing different methods of facilitation (38) and examining the more detailed mechanisms through which facilitation operates (33). However, there are still important questions that need to be considered and addressed, as we outline in the following section.

## Lessons Learned and Future Directions

Cross-case comparisons of facilitation studies highlight a number of important lessons for others considering this approach within an improvement program (23). These include the importance of balancing a structured approach to project management with flexibility and responsiveness to issues that arise during the course of the project. Marshall and colleagues have recently described their experience of trying to design an effective improvement intervention (2). Despite careful attention to the design process, including starting with an initial program theory, only three of the nine components of their planned intervention were implemented in line with the original proposal. This clearly demonstrates the way that complex interventions “morph” and the real challenges involved in tailoring improvement initiatives in real time. Other learning focuses on the need for realistic expectations and allowing sufficient time to see impact, as the study of neonatal mortality in Vietnam illustrated; here, it was not until the third year of the facilitation intervention that significant improvements were observed (37). This same study also demonstrates the importance of selecting people with the right knowledge and skills into the facilitator role (30). Other research highlights relationship building and the need for leadership championing and support (34). While there is no “one-size-fits-all” specification for the facilitator role, there are some guiding principles around the need for creating a supporting environment for facilitators, including mentoring arrangements for those who are new in the role (29).

So where next in terms of facilitation and facilitators in healthcare quality improvement? We would suggest that there are a number of areas that would be beneficial to explore further around the role and its contribution to enabling CQI.

1. Achieving clarity around the core elements of facilitation and clearly defining what comprises (or not) a facilitation intervention. This will help in delineating facilitation from other commonly applied improvement and implementation roles; equally it can guard against the role being inappropriately described or applied (39).
2. Linked to issues around role clarity, we need to become more adept at finding, selecting, and preparing the right people to take on the facilitator role.
3. We need to know more about how to build the right networks to support and mentor facilitators—understanding what facilitator “chains” (40) are required to maximize the impact and sustainability of quality improvement programs in healthcare.
4. There are questions related to the cost-effectiveness of facilitation. Clearly, it is a resource-intensive strategy and there is some evidence that “more” facilitation produces greater improvement (36); however, issues relating to the dose, frequency, and intensity of facilitation are important to address to optimize the return on investment.
5. We suggest that we need more studies that build a rounded, comprehensive, and nuanced understanding of the practice of facilitation. Baskerville and colleagues concluded their systematic review with the statement that practice facilitation can work, despite varied challenges. As such, they suggested that further randomized controlled trials to test facilitation would add less new knowledge, compared to large-scale, collaborative practice-based evaluation research. The latter could help to understand the mechanisms of facilitation, including relationships between context and components of facilitation, and issues relating to sustainability and costs to the health system (36).

Further advancing our understanding of facilitation processes and facilitator roles will help to improve the prospective design and application of improvement interventions and our ability to tailor interventions to context.

## Author Contributions

GH and EL equally contributed to the planning and writing of the manuscript; both reviewed and approved the final submission.

## Conflict of Interest Statement

The authors declare that the research was conducted in the absence of any commercial or financial relationships that could be construed as a potential conflict of interest.
